# 

**Published:** 1845-01

**Authors:** 


### MEDICO-CHIRURGICAL

### REVIEW,

AND

### JOURNAL OF PRACTICAL MEDICINE.

v. [42]

NEW SERIES.

**Vol. I.**

OCTOBER 1, 1844, TO MARCH 31, 1845.

Surgeon Genl's Office
LIBRARY.
Washington, D.C.
57442

LONDON:

SAMUEL HIGHLEY, FLEET STREET.

1845.

W1
ME 75T

PRINTED BY F. HAYDEN,
Little College Street, Westminster.

### INDEX.

A.

Abortion induced by scrofula ...... 269
Abscess, intrapelvic, Marchal on .... 134
Abscess, intrapelvic, seat of ........ 135
Abscess, intrapelvic, symptoms and diagnosis ..................... 141
Abscess, intrapelvic, etiology ...... 142
Abscess, intrapelvic, in males ...... 143
Abscess, ilio-cœcal .. ............ 146
Abscess, observations by Miller on .. 245
Abscess, milk, prevention of ...... 384
Absorbents, their agency in ulceration 104
Acton's modern cookery .......... 560
Adams' translation of Paulus Ægineta 189
Adhesion, Travers on the process of.. 101
Ague, Lefevre on treatment of ...... 374
Air, exclusion of in the treatment of disease........................ 383
Air-cells, structure of.............. 330
Aldis', Dr. evidence before Towns' Commission ............ ..... 113
Algeria, medical history of.......... 199
America, state of medical science in.. 366
Ammoniæ aqua ... .............. 207
Ammoniæ acetatis aqua .......... 210
Ammoniæ bicarbonas ............ 212
Ammoniæ spiritus ................ 212
Amputation, entrance of air into the veins during .................. 11
Amputation, treatment of stumps after 56
Anatomical sketches and diagrams .. 185
Anatomy, Lizars' text book of ...... 484
Anatomy, Serres on transcendental .. 168
Anatomy, comparative, illustrating human organogeny.............. 177
Aneurism of the aorta ............ 439
Aneurism of the external iliac ...... 442
Aneurism, observations on ........ 343
Aneurism, history of the operation for 345
Aneurism, Anel's operation for...... 346
Aneurism, Hunter's operation for.... 349
Animalculæ, spontaneous generation of.......................... 569
Animals, Jones' natural history of .. 520
Antidotes, Christison and Orfila on .. 457
Antimonii oxydum................ 212
Antimonium tartarizatum.......... 213
Apology for the nerves ............ 367
Apoplexy, prevention of .......... 384
Apothecaries' act, defects of........ 287
Apothecaries' Society, address of 277, 561
Apprenticeship, studies during ...... 87
Armies, proportion of sick in ...... 197
Arsenic, poisoning by.............. 470
Arsenic, poisoning by, tests of ...... 471
Arsenic, poisoning by, action and symptoms of .................... 473
Arsenic, poisoning by, morbid appearances from .................... 475
Arsenic, poisoning by, treatment of.. 475
Arsenic, poisoning by, diuretics in .. 476
Arsenicalis, liquor ................ 215
Arteritis, chronic, Tommasini on.... 300
Artery, pulmonary, obstruction of the branches of.................... 425
Artery, common iliac, ligature of .... 442
Ashwell on diseases peculiar to women 32
Assimilation, Liebig and Mulder's theories of .................... 527
Atmosphere, temperature of ........ 418
Atmosphere, humidity of .......... 419
Atmosphere, weight of ............ 420

B.

Ballingall, Sir G., on military surgery 193
Barlow's, Dr. clinical reports ...... 69
Barlow, Rev. J. on self-preservation from insanity ................. 519
Bartlett's, Dr. philosophy of medical science........................ 353
Barytæ muriatis solutio............ 216
Barytæ nitras .................... 507
Bed-sores, treatment of............ 249
Bell's, Sir Charles, anatomy and philosophy of expression............ 220
Berutti on spontaneous generation . 569
Bile discharged from a tumour in hypochondrium .................. 447
Bird, Dr. Golding, on urinary deposits 526
Births, proportion of male to female 341
Bismuthum album................ 217
Bladder, fistulous communication with the ilium...................... 450
Blood, influence of the colourless corpuscules ...................... 335
Blood, composed of floating cells.... 336
Blood, pathological changes in ...... 337
Blood, philosophy of moving powers of the ........................ 555
Blood, difference of composition in the sexes........................ 582
Bloodletting, Dunglisson on ........ 87
Bloodletting in inflammation, Travers on ............................ 95
Bone, development of ............ 570
Bone, inflammation of ...... .. 89, 251
Brain, white softening of, from aneurism of aorta .................. 439
Brain, hydatids of ................ 4
Brain, case of insidious disease of .. 202

Brain, prevention of disease of, in children ........................ 388
Brain, Ramolissement in the aged .. 125
Britain, state of medical science In .. 365
Bronchi, tubular expectoration from 443
Bronchial glands, lesions in phthisis.. 399
Bronchitis, position of organs in.. .. 47
Bronchitis. chronic, treatment of..... 387
Broussais, critique upon, by Bartlett 363
Brown on asthma and consumption.. 416
Burn, case of extensive............. 159
Butyric acid in the urine ..... .... 530
C.
Calcis murias...................... 218
Capillaries, structure and functlons of 92
Capillaries, their influence upon the circulation . .................. 557
Carbon, deposition of in the lungs .. 571
Carcinoma of the thyroid gland ....8, 10
Carcinoma of the lungs............ 28
Carcinoma, colloid................ 447
Carcinoma, intimate structure of.... 567
Carpenter's principles of human physiology........................ 321
Cartilage, non-vascularity of........ 338
Caustic, Cazenave on various kinds of 577
Cell-theory of nutrition.......... .. 333
Cell-theory of nutrition exemplified 525
Cerebral diseases, causes of ........ 123
Cerebral diseases, treatment of.. .... 125
Cerebritis, paralytic ............... 120
Chemistry, Fownes', manual of elementary ........................ 186
Chest, chronic diseases, treatment of 387
Chick, process of development of the 173
Children, respiratory movements of.. 46
Children, diet of. ................ 230
Children, convulsions of .......... 381
Chlorosis, treatment of............ 576
Cholera, observation by Larrey upon 61
Chorea, shower-bath in.......... . 327
Christison's, Dr. dispensatory .. 204, 489
Christison's, Dr. treatise on poisons.. 451
Cicatrization, Travers on .......... 105
Circulation, Dr. Calvert Holland on.. 555
Clothing, military, observations upon 195
Coagula, organization of in cachexia.. 16
Cold, effects of extreme, in Russia .. 368
Constipation, cases of.............. 70
Constipation, nauseants in ... .... 85
Constipation, strychnia in...... ... 185
Contusions, warm local applications in 158
Cookery, Acton's modern .......... 560
Cooper's, Mr. B., lectures on osteology 87
Cooper's, Mr. B., case of admission of air into the veins .............. 11
Cooper's, Mr. B., case of extirpation of ovarian cyst ................ 18
Cornea, transplantation of........... 572
Cornea, non-vascularity of ........ 338
Corpus luteum, Ritchie on varieties of 341
Corrosive sublimate, poisoning by .. 47[illegible]
Corrosive sublimate, tests of purity of 49[illegible]
Creta preparata ................... 2[illegible]
Cupping, Marshall Hall on ........ 3[illegible]
Cysticercus cellulosa of the brain....
Cystine, or cystic oxide in the urine.. 5[illegible]
D.
Dalrymple on organization of coagula [illegible]
Davy on composition of the meconium 4[illegible]
Dentition, scarification of the gums in 3[illegible]
Development, Serres on the laws of.. 17[illegible]
Development, centripetal .......... 1[illegible]
Development, gradations of tissue in 1[illegible]
Development illustrated by monstrosities .......................... 1[illegible]
Diagnosis, Bartlett on ............ 3[illegible]
Diarrhœa, fibrinous .............. 1[illegible]
Dictionary of terms used in medicine 2[illegible]
Diet of soldiers .................. 1[illegible]
Diet, Parry on..................... 2[illegible]
Digitalis, Munk on the administration of [illegible]
Diseases, classification of .......... 3[illegible]
Diseases from spurious organization.. 5[illegible]
Dispensatories, Christison's and Thomson's .................... 204, 4[illegible]
Diuretics, administration of ........ 5[illegible]
Dracunculus, case of............... 57[illegible]
Duality a law of development ...... 17[illegible]
Duality of the mind ............... 51[illegible]
Dunglison's general therapeutics .... 8[illegible]
Electricity, therapeutical use of currents of ....................... 31[illegible]
E.
Electro-physiology, Matteucci on.... 30[illegible]
Embryology, Serres on .... ...... 17[illegible]
Emotions, manifestation of the .... 22[illegible]
Emphysema of the lungs, nature of.. 4[illegible]
Empyema, paracentesis in ... 431, 436
Enemata, mode of employing ...... 85
Enemata, Hall on medical uses of.... 383
Epilepsy, corrosive sublimate in .... 579
Erichsen's, Mr, observations on aneurism.............................. 343
Erysipelas, erratic ................ 299
Evans' lectures on phthisis ........ 395
Expression, Bell on anatomy and philosophy of ...................... 220
F.
Ferri oxidum nigrum ............ 218
Ferri sulphuretum............... .... 489
Ferri sulphas ..................... 492
Ferrugo, preparation of............ 490
Ferrum tartarizatum .............. 494
Fever, Southwood Smith on that of the Metropolis ...................... 109
Fever induced by depressing emotions 373
Fever, typhoid, emetics and purgatives in.......................... 575
Fistulæ, cervical .................. 570
Fistulæ, vesico-vaginal ............ 297
Flourens on development of bone .. 570

Fownes' manual of chemistry ...... 186
Fractures, Larrey's appareil for .... 67
Fractures, partial of neck of thigh-bone ........................ 80
Fractures, Cooper on treatment of .. 89
Fractures, treatment by suspension.. 157
Fractures, local applications to ..... 158
France, state of medical science in .. 365
France, Mr., pathology of iritis .... 79
Friction, Lefevre on the use of...... 371
Frogs, electrical experiments with .. 309
Fulgurites, Kaemtz on ............ 422
Fungus hæmatodes uteri .......... 36

G.

Galvani, dates of his experiments.... 307
Galvanism, medical, Grantham on .. 161
Ganglionic nerves, structure of .... 487
Gangrene, Travers on ............ 106
Gangrene, amputation in .......... 249
Gangrene, hospital ............... 250
Gangrene of the mouth............ 577
Generation, spontaneous .......... 569
Geological formations ............ 148
Girls, hygiene of at puberty........ 581
Grantham, facts and observations .. 157
Grape-cure, Lefevre on ............ 375
Gravel, uric acid, treatment of...... 536
Gravel, white, nature and treatment, 547
Green, Dr. on tubercle in children .. 444
Green, Mr. on medical reform ...... 277
Greenhow's removal of diseased ovarium ........................ 20
Greenhow's case of Miss Martineau, 508
Guy's, Dr., principles of forensic medicine ........................ 458
Guy's, Dr., evidence before towns commission ...................... 112
Guy's Hospital Reports............ 63

H.

Hæmoptysis in phthisis ............ 398
Hail, Kœmtz on production of...... 423
Hall's, Dr. Marshall, practical observations ...................... 377
Harvey's early observations on embryology ...................... 170
Headache, Lefevre on ............ 371
Heart, dimensions of its cavities .... 48
Heart, digitalis in diseases of ...... 77
Heart, case of malformed.......... 575
Hernia, Hewett on omental sacs in strangulated .................. 437
Hippuric acid in urine ............ 530
Hoblyn's Dictionary of Terms ...... 291
Hocken, Dr. on phthisis .......... 164
Holland, Dr. on philosophy of the moving powers of the blood...... 555
Homicidal mania ................ 118
Hooping-cough, Lefevre on ........ 372
Horns, development in the human skin.......................... 13
Hughes on paracentesis thoracis ..... 81
Humerus, spina ventosa of ........ 203
Hutchinson on the capacity of the lungs .......................... 332
Hydrocele, spermatozoa in the fluid of .......................... 5, 7
Hydrocele of the neck ............ 572
Hydrocephalus, pressure in ........ 160
Hydrocyanic acid, poisoning with ... 481
Hydrometra, Ashwell on .......... 39
Hygiene, military ................ 194
Hygiene, public, observations upon.. 108
Hyoid bone, expulsion of .......... 296
Hypochondriasis, Pinel upon....... 117
Hypothesis, Bartlett on .......... 355
Hypothesis, medical .............. 363

I.

Ilium, fistulous communication with the bladder...................... 450
Incendiary mania ................ 119
Inflammation, Travers and Bennett upon .......................... 91
Inflammation, Miller on .......... 239
Inflammation, theory of........ 93, 239
Inflammation, constitutional symptoms .......................... 94
Inflammation, bleeding in ...... 95, 242
Inflammation, terminations and results ....................... 96, 241
Inflammation, local applications .... 243
Inflammation, not elucidated by physiology ........................ 356
Insanity, Pinel on .............. 115
Insanity, causes of.............. 123
Insanity, hereditariness.......... 123
Insanity, therapeutical management.. 126
Insanity, moral and hygienic management ........................ 128
Insanity, non-restraint .......... 132
Insanity, employments and amusements ........................ 133
Insanity, religious services in ...... 133
Insanity, diet in.................. 134
Insanity, moral .................. 120
Insanity, premature seclusion in.... 160
Insanity, Wigan on nature & forms of, 518
Insanity, Barlow on self-preservation from ........................ 519
Instincts, lesions and perversions of.. 116
Intemperance as a cause of disease .. 112
Intestinal irritation, Dr. M. Hall on.. 390
Intestinal villi, structure of ........ 330
Iritis, France on pathology of ...... 79

J.

Jahn on spurious organization...... 564
Jaw, lower, necrosis of ............ 448
Joints, Miller on diseases of ........ 253
Jones, Dr. Bence, on the urine.... 27, 31
Jones', Professor, Natural History of Animals ........................ 520
Jugular, pulsation of, effect of respiration on ...................... 45

K.
Kaemtz' course of meteorology ..... 417
Kidney, supposed rupture of pelvis of, 2
L.
Larrey's medical campaign and travels 49
Laryngitis, diagnosis of............ 394
Lead, effect of upon water.......... 480
Lectures, popular, on science, insufficiency of .................... 521
Lefevre's apology for the nerves .... 367
Light, solar, importance of to health, 111
Lightning, heat, Kaemtz on ........ 422
Lizars' text-book of anatomy ...... 484
Louis' researches on phthisis ...... 395
Lugol on causes of scrofula ........ 258
Lunatic asylums, reports of ........ 115
Lunatic asylums, arrangements proper for ......................... 130
Lungs, carcinoma of............... 23
Lungs, deposition of carbon in...... 571
Lungs, intimate structure of ....... 330
Lungs, capacity of................ 332
Lungs, production of emphysema in, 47
Lupus, cod-liver oil in ............ 579
M.
Magnetism, terrestrial ............ 424
Magnetism, human ............... 508
Malaria at Petersburgh............ 373
Man, geological history of......... 153
Man distinguished from other mammalia ....................... 325
Mania, varieties of................ 119
Marchal (de Calvi) on intrapelvic abscess ......................... 134
Marriage, early, a cause of scrofula .. 265
Matteuci on electro-physiology ..... 305
Meconium, composition of ........ 429
Medicine, evil of hypothesis in...... 362
Medicine, state of in Europe & America 365
Medico-chirurgical transactions ..1, 425
Meningitis, tuberculous............ 403
Menstruation, discharge of ova during ......................340, 580
Mesmerism ..................508, 370
Meteorology, Kaemtz on .......... 417
Military surgery, Ballingall on ...... 193
Military surgery, cases in ....... 62, 202
Milk, abscess and fever, prevention of 384
Miller's principles of surgery ....... 236
Mind, the duality of .............. 543
Moles, uterine, Ashwell on ....... 39
Mollities, ossium, pathology of...... 448
Monstrosities, Serres on .......... 180
Morphia and its salts.............. 497
Mortification, Travers on .......... 106
Mortification, Miller on............ 248
Motion, voluntary, lesions of ...... 120
N.
Naphtha in phthisis .............. 164
Natural history of animals, by Prof. Jones ......................... 520
Necrosis, Miller on .............. 252
Nerve, the sympathetic, Procter on.. 182
Nerves, Bell's respiratory system of.. 221
Nerves, effects of electrical currents on 314
Nerves, motor and sensitive, distinguished by electricity ........... 317
Nerves, quantity of blood supplied to, 327
Nerves, apology for the............ 367
Nerves, the ganglionic ............ 487
Neuropathy, Marshall Hall on...... 506
Newnham on human magnetism .... 508
Nutrition, cell theory of .......... 333
Nutrition, Liebig & Mulder's views on 527
O.
Observation, medical, laws of ...... 361
Ophthalmia, epidemic, Larrey on.... 58
Opium, Taylor on the tests for...... 76
Orfila's treatise on toxicology ...... 451
Organology, laws of .............. 175
Organology, human, illustrated by comparative anatomy ........... 177
Organization, spurious ............ 564
O'Shea on medical reform ......... 561
Ossification, accidental............ 90
Ova, maturation and discharge of during menstruation.......... 339, 580
Ovarian cyst, extirpation, cases of.. 18, 20
Ovarian tumors, Philipps on the operation for the removal of ........ 22
Ovarian tumors, Ashwell on ....... 41
Ovum, anatomy of the ............ 488
Oxalate of lime in the urine .... 31, 544
Oxalic acid, poisoning by .......... 477
Oxalic acid, frequent existence in urine 542
Oxalic acid, abundance of in plants.. 543
Oxygen, history and sources of ..... 187
P.
Paget on obstruction of the pulmonary artery ......................... 425
Pain, expression & representations of, 225
Paracentesis thoracis ...... 81, 431, 436
Paralysis, general ................ 120
Paralysis, electric currents applied for 520
Parry on diet .................... 229
Pathology, study of, independent of physiology ..................... 358
Paulus Æginata, Adams' edition of .. 189
Pelvis, abscess within the.......... 135
Peritonitis, tuberculous ........... 402
Pharmacopœia, the Edinburgh.. 204, 489
Philipps on the operation for ovarian tumors ........................ 22
Phrenology, Lefevre on............ 369
Phthisis, Hocken on naphtha in .... 165
Phthisis, Evans and Louis on ...... 395
Phthisis, prevalence in different classes 112
Phthisis, pathology of ............ 396
Phthisis, identity with scrofula denied 397
Phthisis, hæmoptysis in .......... 398
Phthisis, lesion of bronchial glands in 399
Phthisis, semeiology of ........... 402

Phthisis, chronic peritonitis in...... 402
Phthisis, meningitis in ............ 403
Phthisis, diagnosis of.............. 404
Phthisis, prognosis and curability of 408
Phthisis, etiology of .............. 409
Phthisis, treatment of ............ 411
Phthisis, counter-irritation in ...... 414
Phthisis, application of lotions in 379, 416
Phthisis in children................ 444
Phosphates in the urine, treatment of 547
Physiology, transcendental, Serres on 168
Physiology, Carpenter's principles of human .......................... 321
Physiology, improved modern mode of study of ........................ 321
Physometra, Ashwell on ........... 38
Pinel on cerebral pathology ........ 115
Plague, causes of in Egypt ........ 293
Pleurisy, cataplasms in ............ 383
Plica polonica, nature of .......... 337
Plumbi acetas...................... 499
Plumbi nitras ..................... 499
Pneumonia, primary seat of ........ 47
Poisoning, treatment of............. 457
Poisoning, evidence of ............. 458
Poisoning, value of symptoms of,460, 474
Poisoning, morbid appearances of.... 462
Poisoning, analysis in.............. 462
Poisoning, experiments on animals .. 465
Poisoning, moral evidence of........ 468
Poisoning, defective investigation by coroner's juries ................ 465
Poisons, Christison and Orfila on.... 451
Poisons, modus operandi of ........ 451
Poisons, absorption of ............ 452
Poisons discovered in the tissues .... 453
Poisons, causes modifying action of .. 455
Poisons, effects of habit and idiosyncracy in reference to ............ 456
Poisons, quantity detected by analysis not great........................ 464
Poisons, imbibition of ............ 467
Polypus uteri, Ashwell on.......... 33
Potassa aqua ..................... 501
Potassæ acetas ................... 500
Potassæ carbonas ................. 501
Potassæ and sodæ tartras .......... 502
Potassii sulphuretum............... 503
Procter on the sympathetic ........ 182
Prognosis, Marshall Hall on ........ 386
Propensities, lesions and perversions of 116
Prostitution, singular institution of in Algeria ......................... 199
Puerperal women, intrapelvic abscess in 136
Pulmonary artery, obstruction of the branches of..................... 425
Pulse, the venous ............ 295,557
Purpurine of the urine............ 539
Pus, Travers on formation of ...... 101
Pyne on vital magnetism .......... 508

R

Rainey on structure of the lungs .... 330
Reasoning, inductive, Bartlett on 354,359
Reform, medical .............277, 561
Respiratory movements in children.. 46
Rigby, evidence before towns' Commission ........................ 114
Roe on paracentesis thoracis........ 433
Scirrhus, intimate structure of...... 568

S

Science, physical, Bartlett on ...... 353
Science, medical philosophy of...... 356
Science, medical, independence of its various branches ................ 358
Science, medical, its prospects ...... 365
Scrofula, Lugol and Smith upon .... 258
Scrofula, hereditary transmission of 261,272
Scrofula, identity with tubercle 263, 397
Scrofula, influence of early marriage in 265
Scrofula, treatment of ............ 273
Sebaceous glands, pathology of the .. 13
Secale cornutum, Grantham on .... 162
Sensation, definition of ............ 327
Sensation special, not transferable .. 328
Sensation, lesions of .............. 122
Serres, transcendental physiology of.. 168
Setons, Marshall Hall on ......... 382
Sexes, proportion born of .......... 341
Sewerage, defects of metropolitan, 114,115
Shock, state of ................... 244
Sibson on changes in the situation of organs ......................... 44
Sinking, state of .................. 392
Sirocco, influence of on health...... 201
Skeleton, comparative anatomy of .. 324
Skin, classification of diseases of the 292
Smith, Dr. Southwood, evidence before Town's Commission ............ 109
Smith, Dr. Tyler, on scrofula ...... 258
Sodæ carbonas .................... 504
Sodæ phosphas ................... 505
Soldiers, hygiene of ............... 194
Soldiers, diseases of ............... 197
Solly on mollities ossium .......... 448
Spermatozoa in hydrocele .......... 5, 7
Spermatozoa in urine............... 552
Spinal cord, functions of .......... 486
Sterility, Marshall Hall on.......... 385
Sterility, strychnia in.............. 184
Storms, causes of ................. 420
Sugar, detection of in urine ........ 554
Suicide of the insane.............. 117
Suppuration, Travers on .......... 101
Surgeons, College of, disputes in the.. 285
Surgery, Miller's principles of ...... 236
Sydenham Society, publications of the ................ 189, 343, 395

T

Tartaric acid ..................... 206
Taylor's thermometrical table ...... 559

Therapeutics, Bartlett on principles of, 359
Therapeutics of nature ............ 294
Thermometrical tables, Taylor's .... 559
Thigh-bone, partial fracture of neck of, 80
Thomson's dispensatory........ 204,489
Throat, disease of, tracheotomy in .. 30
Thumb, luxation of ............. 297
Thymus gland, uses of ........... 296
Thyroid gland, carcinoma of ...... 8, 10
Tissues, non vascular ........... 338
Tissues, gradation of ............. 179
Tophi, Miller on................. 253
Towns, health of, commission ...... 108
Tracheotomy in disease of the throat 30
Travers on physiology of inflammation 91
Trephine, Cooper on the use of...... 90
Tubercle, question of identity with scrofula................. 263, 397
Tubercle, seat of in children........ 444
Tumours, Miller on .............. 256

U

Ulceration, Travers on ............ 104
Ulcers, treatment of........... 105,247
Unguentum citrinum ........... ... 495
Urea, origin of .................. 529
Ureter, supposed rupture of ........ 2
Uric acid, origin of, in the urine .. 27, 529
Uric acid, treatment when in excess.. 536
Uric oxide in the urine ............ 539
Urinary deposits, Bird on the classification of ..................... 531
Urine, Bird on deposits in.......... 526
Urine, chemical composition of....... 528
Urine, clinical examination of ...... 532
Urine, physiological origin of ...... 527
Urine, blue and black............. 549
Urine, blood and other organic deposits in ........................ 550
Urine, detection of sugar in ........ 552
Uterine hæmorrhage, application of the child in.................... 386
Uterus, polypus of................ 3[illegible]
Uterus, malignant growths and ulcers within the ................... 36[illegible]
Uterus, fungus hæmatodes of ...... 36[illegible]
Uterus, ulceration of mucous membrane of ..................... 36[illegible]
Uterus, tympanitis of ........... 38[illegible]
Uterus, dropsy of ............... 39[illegible]
Uterus, hydatids in .............. 40[illegible]

V

Vaccination, premature............ 162
Veins, admission of air into, during amputation .................. 11
Veins, pulsation in............ 295,557
Vernix caseosa, composition of...... 430
Vesico-vaginal fistula.............. 297
Vestiges of natural history of creation 147
Vision, complex nature of sensation of 329

W

Wigan on the duality of the mind.... 513
Wilson on pathology of the sebaceous glands ...................... 13
Winds, Kaemtz on the production of 419
Women, Ashwell on the diseases of.. 33
Wormald's anatomical sketches...... 185
Wounds, Larrey on healing of .... 55,67
Wounds, Travers on the healing of .. 100

### BIBLIOGRAPHICAL RECORD.

1. Lectures on Osteology, including the Ligaments which connect the Bones of the Human Skeleton. By B. B. Cooper, F.R.S., Surgeon to Guy's Hospital, &c. &c. &c. 8vo. pp. 272. Highley, Fleet-street, 1844.

2. Guy's Hospital Reports. Second Series, No. 4, October, 1844. Edited by Drs. Barlow, Cock, Birkett, Browne, and Poland. Highley, 1844.

3. The Ethnology of the Ancient Irish. By W. R. Wilde, M.R.I.A. 8vo. pp. 16. Dublin, 1844.

4. Contributions to Ophthalmic Surgery.—Causes and Treatment of Entropicus and Trachiasis. Also, by the same Author: Contributions to Aural Surgery, &c. 8vo. pp. 36. Dublin, 1844.

5. The Pharmaceutical Journal, Vol. IV. No. IV. October, 1844.

6. The Northern Journal of Medicine. No. VI. October, 1844.

7. Sir James Graham's Bill repudiated. By John Thomson, M.D. Being "Annals of Medicine," No. 1. Highley, October, 1844.

8. Phrenological Journal, Vol. XVII. No. 81. Quarterly. 1844.

9. The Anatomy of the Arteries. By Richard Quain, Esq. F.R.S. Part XVII. Seven Plates, with Letter-press. Price 20s. Folio. Taylor and Co. Gower-street.

10. The Principles of Surgery. By James Miller, F.R.S. Esq. Professor of Surgery in the University of Edinburgh, Surgeon to the Royal Infirmary, &c. Small 8vo. pp. 712, with Index. Adam and C. Black, Edinburgh. Longman's. London, 1844.

11. Introductory Address to the Students of University College, 1st of October, 1844. By Samuel Cooper, Professor, &c. Longman and Co. 1844.

12. First Report of the Commissioners for Enquiring into the State of Large Towns and Populous Districts. Vols. 1 and 2. Oct. 1844.

13. Facts and Observations in Medicine and Surgery, &c. By John Grantham, Fellow of the Royal Col. of Surgeons, &c. 8vo. Churchill, 1844.

14. Oulines of Military Surgery. By Sir George Ballingall, M.D. F.R.S.E. Third Edition, 1844.

15. Vestiges of the Natural History of Creation. 8vo. pp. 390. Churchill, 1844.

16. Principles of Forensic Medicine. By William A. Guy, M.D. Fellow of the Royal College of Physicians, Professor of Forensic Medicine, King's College, London. Part III. Renshaw, Strand, Oct. 1844.

17. A Practical Enquiry into the Value of Medicinal Naphtha in Tubercular Phthisis. By Ed. Oct. Hocken, M.D. 8vo. Highley, 1844.

18. An Exposition of the Laws which relate to the Medical Profession in England, &c. &c. By John Davies, M.D. Highley, 1844.

19. An Inaugural Lecture on Chemistry. By Thos. George Titley, Professor of Chemistry in Queen's College, Birmingham. Van Voorst. London, 1844.

20. The Dublin Journal of Medical Science for November, 1844. *In Exchange.*

21. The Northern Journal of Medicine. No. VII. Nov. 1844.

22. The London and Edinburgh Monthly Journal of Medical Science. Nov. 1844.

23. Dictionary of Terms used in Medicine, and the Collateral Sciences. By RICHARD HOBLYN, A.M. Oxon, Author of a Manual of the Steam Engine—a Manual of Chemistry, &c. 8vo. Second Edition, revised and enlarged. pp. 394, Double Columns. Sherwood and Co. Dec. 1844.

24. An Address by the Society of Apothecaries to the General Practitioners of England and Wales on the Provisions of Sir James Graham's Bill, &c. &c. Highley, 1844.

25. On Diet, with its Influence on Man; being an Address to Parents, &c. or how to Obtain Health, Strength, Sweetness, Beauty, Development of Intellect and Long Life. By THOMAS PARRY. Octavo, pp. 119. Highley. Nov. 1844.

26. Sequel to Homœopathy Unmasked. By ALEXANDER WOOD, M.D. Edinburgh. Nov. 1844.

27. On the Changes Induced in the Structure and Situation of the Internal Organs under varying Circumstances of Health and Disease, &c. By FRANCIS SIBSON, Resident Surgeon to the General Hospital, Nottingham. 8vo. pp. 574. Deighton, Worcester, 1844.

28. Medical Reform; a Letter from Charles T. Carter, Esq. to the Secretary of the North of England Medical Association.

29. The Cyclopædia of Anatomy and Physiology. Edited by ROBT. B. TODD, M.D. F.R.S. Part XXVI. Nov. 1844. Sherwood and Co.

30. A New View of Insanity. The Duality of the Mind proved by the Structure, Functions, and Diseases of the Brain, &c. &c. By A. L. WIGAN, M.D. 8vo. pp. 459. Longman and Co. 1844.

31. An Apology for the Nerves: or their Influence and Importance in Health and Disease. By Sir GEO. LEFEVRE, M.D. 8vo. pp. 363. Longman and Co. 1844.

32. Lectures on Pulmonary Phthisis, Delivered in the Jervis-street, Hospital. By JOHN EVANS, M.D. 8vo. pp. 193. Longman and Co. Nov. 1844.

33. History of British Crustacæa. By THOMAS BELL, F.R.S. &c. Part I. Oct. 1. 1844. Price 2s. 6d. Van Voorst. Nov. 1844.

34. The Medical Examiner, and Record of Medical Science. Edited by Dr. HUSTON. Nos. 16, 17, 18. Philadelphia, 1844. *In Exchange.*

35. Obstetric Practice, Report of, from the University College Hospital. By ED. W. MURPHY, M.D. A.M. Professor of Midwifery in the College. 8vo. pp. 54. Dublin, 1844.

36. An Address read to the Harveian Society. By ED. W. MURPHY, M.D. &c. pp. 16. Taylor and Walton, 1844.

37. Londres et Les Anglais, des Temps Modernes. Par le Docteur BUREAUD RIOFREY. Bailliere, Londres, 1844.

38. The American Journal of Insanity. Edited by the Officers of the New York State Asylum. Oct. 1844.

39. Pathologia Indica; or the Anatomy of Indian Diseases, Medical and Surgical. By ALEXANDER WEBB, B.M.S. Part I. Medical Pathology. Calcutta, 1844.

40. American Journal of the Medical Sciences. Edited by ISAAC HAYS, M.D. October, 1844.

41. An Essay on the Philosophy of Medical Science. By ELISHA BARTLETT, M.D. Philadelphia, 1844.

42. A Trestise on Poisons in relation to Medical Jurisprudence, Physiology, and the Practice of Physick. By ROBERT CHRISTISON, M.D. &c. &c. Fourth Edition, November, 1844. A. Black, Edin.; Longman's, London.

43. Remarks on the Present State of the Medical Profession in this Country, &c. By THOMAS NUNNELEY, Esq. 1844.

